# A Field Emission X-Ray Source Array for Stationary Digital Chest Tomosynthesis Applications

**DOI:** 10.3390/s26113592

**Published:** 2026-06-05

**Authors:** Huaping Tang, Fengyan Zhang, Guoyu Li, Biao Wang, Wu He, Runze Fang, Zhiqiang Chen

**Affiliations:** 1 NuRay Technology Co., Ltd., Changzhou 213000, China; 2 Department of Engineering Physics, Tsinghua University, Beijing 100084, China

**Keywords:** carbon nanotube, field emission cathode, high mAs, multi-beam X-ray source, stationary digital chest tomosynthesis

## Abstract

Digital chest tomosynthesis (DCT) has been clinically validated to offer significant advantages in diagnostic efficiency for pulmonary diseases and radiation dose reduction. Emerging stationary DCT (sDCT) systems can further shorten acquisition time and eliminate motion artifacts caused by X-ray source movement and patient respiration. This work focuses on the development of a multi-beam X-ray source for mobile sDCT systems by specification definition, source design, and experimental validation. The developed X-ray tube integrates 63 focal spots arranged linearly over a length of 816 mm. X-rays are emitted through seven segmented windows, achieving an angular span of 36° at a source image distance (SID) of 120 cm, with full coverage of a detector area of 35.6 cm × 43.2 cm. The tube operates at a maximum anode voltage of 140 kV, maximum anode current of 20 mA, and 24 mAs per scan, with a focal spot size of IEC 0.6. The developed multi-beam X-ray source achieves multiple key performance breakthroughs and provides an alternative source architecture for future sDCT implementation, with the potential to facilitate further system performance optimization and engineering development.

## 1. Introduction

**Figure 1:**
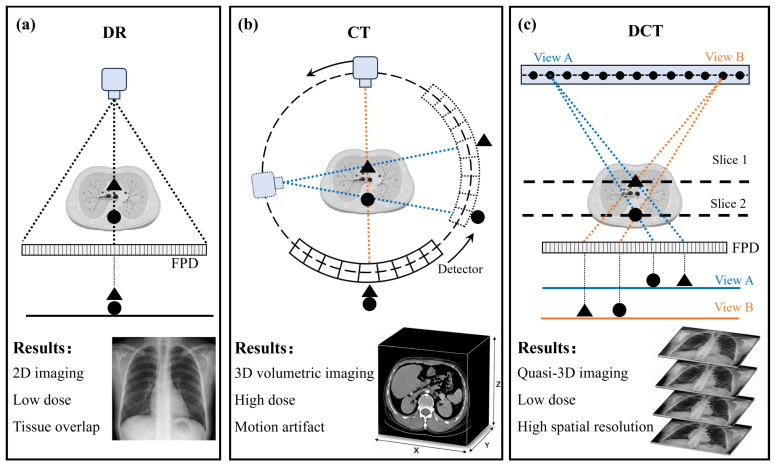
Imaging principles and representative results of (**a**) DR; (**b**) CT; and (**c**) DCT.

Chest X-ray examinations are widely used for the early intervention and management of thoracic trauma and chronic pulmonary diseases [1,2,3]. The primary imaging modalities include digital radiography (DR) and computed tomography (CT). DR compresses three-dimensional anatomical structures into a two-dimensional projection, resulting in tissue overlap that can obscure lesions (false negatives) or create superimposed structures mimicking pathology (false positives) [4,5]. CT provides superior diagnostic performance compared with DR [1,6]; however, its relatively long acquisition time and higher radiation dose [7,8] limit its suitability for longitudinal monitoring. Digital chest tomosynthesis (DCT) has emerged to address these limitations. It acquires a series of low-dose projection images over a limited angular range using continuous or step-and-shoot X-ray tube motion, followed by tomosynthesis reconstruction to generate a stack of slice images parallel to the detector plane. DCT has demonstrated improved detection of pulmonary nodules, calcifications, vasculature, airways, and chest wall abnormalities [9,10,11,12,13]. The imaging methods and typical imaging results of DR, CT, and DCT are shown in Figure 1.

DCT combines key advantages of both DR and CT: (1) high spatial resolution enabled by small pixel sizes of flat-panel detectors; (2) improved diagnostic efficiency through quasi-3D reconstruction from multi-angle projections; and (3) a substantially lower radiation dose, approximately 10% of CT [8,9,10], making it suitable for longitudinal follow-up and early screening of diseases such as lung cancer, where it has shown distinct clinical value [4,9,10,11,12,14]. Consequently, DCT has been extensively investigated for early detection, disease assessment, treatment planning, and therapy monitoring of lung cancer, pulmonary nodules, interstitial lung disease, pneumonia, and pleural effusion (Jung et al. [15], J. H. Kim et al. [16], Hartman et al. [17], E. Y. Kim et al. [18], Lee et al. [19], Tongkum et al. [20], Baratella et al. [21], Meltzer et al. [22], Inscoe et al. [23], Diniz et al. [24], Tang et al. [25]). The technology continues to mature and has become a powerful tool for the detection, diagnosis, and management of pulmonary diseases [14,26].

Most current DCT systems employ a moving X-ray source for multi-angle acquisition [4,7,26,27]. Continuous motion during exposure introduces focal spot blur, degrading spatial resolution [14,27]. Alternatively, a step-and-shoot acquisition mode, similar to that used in digital breast tomosynthesis, results in prolonged total scan times and limited repositioning accuracy [14,28]. In addition, involuntary patient motion during acquisition increases the risk of motion artifacts [14,29]. Stationary digital chest tomosynthesis (sDCT) based on multi-beam X-ray sources has recently been proposed [30,31,32]. By eliminating source motion, sDCT reduces scan time and motion artifacts, thereby improving image quality and lesion detectability. The multi-beam X-ray source is the key enabling component, integrating multiple focal spots within a single vacuum envelope and generating X-rays independently from different physical positions via electronic control. As a result, angular scanning can be achieved without mechanical motion of the X-ray source or detector, thereby eliminating motion artifacts while providing higher temporal resolution.

Among emerging multi-beam X-ray source technologies [33], carbon nanotube (CNT) field emission cathodes are particularly attractive due to their compactness, ease of array integration, and simplified thermal management [34]. In a recent review, Mitchell M. Goodsitt et al. [14] suggested that CNT-based multi-beam X-ray sources represent a significant advancement for DCT. Qian et al. [35] at Duke University and UNC developed a novel hybrid type of CNTs, few-walled CNTs (FWCNTs), by improving the synthesis of double-walled CNTs. FWCNTs consist of 2–5 coaxially rolled graphene layers, with diameters ranging from 2 to 10 nm, combining low field emission thresholds with good structural stability; this makes them ideal for achieving high emission current and long-term stability.

Professor Otto Zhou’s lab at the University of North Carolina (UNC) and collaborators at Carestream Health [36] first demonstrated the feasibility of sDCT using a CNT-based multi-beam X-ray source, while identifying limitations of the initial prototype (80 kV anode voltage, 5 mA tube current, and 2.5 mm focal spot) and proposing improvements including higher voltage and current, smaller focal spot size, and a larger angular span. Subsequently, Park et al. at Kyung Hee University [37] developed an improved CNT-based multi-beam X-ray source with higher anode voltage and increased angular coverage; however, the tube current (3.5 mA) remained insufficient for rapid imaging. Our team at NuRay Technology Co., Ltd. (Changzhou, China) previously developed a multi-beam X-ray source achieving 160 kV and 15 mA [38] and investigated radiation field characteristics [39] and shielding design [40], providing useful guidance for sDCT system design. However, its focal spot size (>1.5 mm) was not suitable for sDCT applications. More recently, Alex Billingsley et al. [32] reported second-generation sDCT progress at UNC using a CNT-based multi-beam X-ray source developed by Tang et al. at NuRay Technology Co., Ltd. [25], achieving 120 kV, 20.4 mA, and a 35° angular span. Nevertheless, the mAs remains lower than that of clinically deployed DCT systems from GE Healthcare [41] and Agfa [42], and the focal spot size remains relatively large, indicating the need for further optimization.

In this study, we focus on the development of a multi-beam X-ray source for sDCT applications, aiming to support fast, wide-angle thoracic imaging with high spatial resolution. A next-generation multi-beam X-ray source is proposed based on system-level requirements, including higher anode voltage, higher tube current, small focal spot size, and sufficient angular coverage. A CNT field emission cathode-based multi-beam X-ray source is then designed and fabricated accordingly. Experimental results demonstrate a maximum anode voltage of 140 kV, maximum anode current of 20 mA, maximum pulse width of 20 ms, 0.4 mAs per focal spot, and 24 mAs per scan, with a focal spot size of IEC 0.6. These specifications align with current DCT device operating requirements. This work provides a practical reference for the design of advanced multi-beam X-ray sources and establishes key hardware support for stationary, fast, high-resolution, and wide-angle chest tomosynthesis imaging systems.

## 2. Methods and System Analysis

DCT systems have been clinically approved and deployed, such as the GE Definium 8000 (GE Healthcare, Chicago, IL, USA) [40] and Shimadzu SONIALVISION (Shimadzu Corporation, Kyoto, Japan) [43]. These systems are largely derived from DR platforms and are associated with large footprints, relatively long acquisition times, and the need for breath-holding to mitigate motion artifacts. To address these limitations, new sDCT system concepts have been proposed by UNC and others [17,30,31], in which the X-ray source is the key determinant of system performance. Accordingly, this study starts from the physical design of an sDCT system and derives the technical requirements for a multi-beam X-ray source.

### 2.1. System Architecture

The sDCT approach employs a multi-beam X-ray source in combination with a high-frame-rate flat-panel detector to enable rapid multi-angle image acquisition. The multi-beam X-ray source integrates multiple focal spots that are sequentially activated via electronic control [25], allowing projection imaging from different positions (angles) without mechanical motion between the source and the patient, thereby enabling truly stationary tomosynthesis imaging.

**Figure 2:**
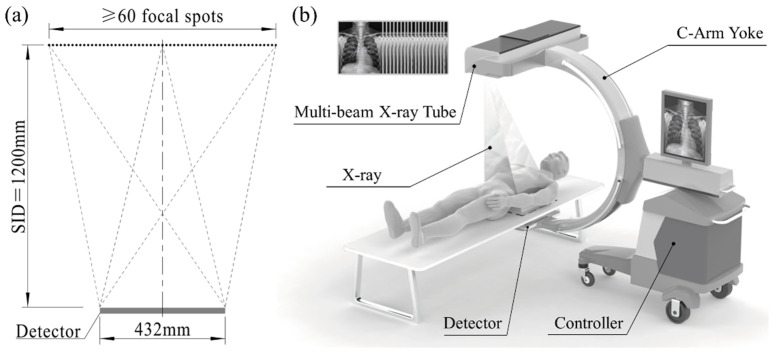
Schematic design of the sDCT system. (**a**) Physical system design; (**b**) structural design.

From a system design perspective, eliminating source motion simplifies the overall structure. First, to accommodate patients with limited mobility, a horizontal (supine) configuration is adopted [27,32]. Second, without mechanical motion constraints, the source-to-image distance (SID) can be reduced. Referencing 110 cm (Shimadzu [43]) and 130 cm (UNC [32]), an SID of 120 cm is selected to balance patient comfort and system compactness. Third, regarding the number of views and angular coverage, Goodsitt et al. [14] reported that clinically deployed DCT systems typically use 60 views with a tomosynthesis angle of 27–40°. In this work, at least 60 views and a tomosynthesis angle of ≥36° are targeted to improve depth resolution and reduce effective slice thickness. A detector size of 35.6 cm × 43.2 cm is adopted. The focal spots in the multi-beam X-ray source are arranged parallel to the patient table, as illustrated in Figure 2.

### 2.2. Requirements for the Multi-Beam X-Ray Source

Key parameters for DCT include anode voltage, focal spot size, and mAs. The typical anode voltage range is 80–140 kV, with values rarely exceeding 120 kV in practice [8,14,32]. Medical X-ray tubes generally have focal spot sizes of 0.8–1.2 mm (IEC 0.6–0.8). Accordingly, the nominal anode voltage in this study is set to 120 kV (up to 140 kV), with a focal spot size of ≤1.0 mm × 1.2 mm, compliant with IEC 0.6. Clinical studies indicate that the total radiation dose for DCT is approximately 2–3 times that of DR [4,5,10], implying low exposure per focal spot (i.e., low mAs). Duke University [8] used 60 views with a total exposure of 30 mAs; reported clinical values include 15 mAs (GE), 2.96 mAs (Shimadzu), and 16 mAs (Agfa, Mortsel, Belgium) [42]. In this study, the total exposure is set to a maximum of 24 mAs, with up to 0.4 mAs per focal spot, reaching the upper range of current clinical practice and representing a substantial improvement over the second-generation sDCT source reported by Alex Billingsley et al. [32].

**Figure 3:**
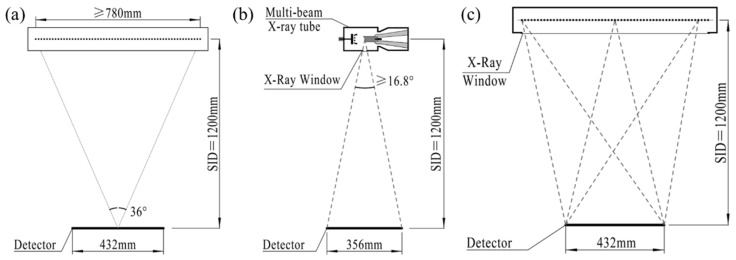
Angular analysis of the multi-beam X-ray tube. (**a**) Schematic of focal spot distribution; (**b**) cone angle direction; (**c**) fan angle direction.

Based on system requirements, SID = 120 cm, detector size 35.6 cm × 43.2 cm, ≥60 views, and angular coverage ≥ 36°; the linear extent of focal spot distribution is designed to exceed 780 mm (Figure 3a). The cone angle for each focal spot (Figure 3b) is designed to be ≥16.8° to fully cover a detector width of 35.6 cm. The fan angle (Figure 3c), aligned with the focal spot array, is implemented via elongated X-ray windows to ensure full coverage of a detector length of 43.2 cm.

To reduce the spacing between focal spots and accommodate a greater number of spots within a limited length, thereby providing more projection angles, enhancing tomosynthesis depth resolution, and reducing effective slice thickness [28] for more accurate diagnosis, this study leverages the high-density arrangement capability of CNT field emission cathodes. By incorporating a tailored focusing structure, sufficiently small focal spot sizes are achieved to meet clinical imaging requirements. Considering the space required for the focusing structure, the inter-focal spot distance was designed to be 12 mm. Based on these considerations, the target parameters of the sDCT system are defined and compared with representative systems, including Agfa DR-600 (Agfa, Mortsel, Belgium) [42,44], Carestream DRX-Evolution Plus (Carestream Health, Rochester, NY, USA) [45,46], Fujifilm FDR Visionary Suite (Shimadzu Corporation, Kyoto, Japan) [47], GE Definium 8000 [41], Shimadzu SONIALVISION [43], Duke (Duke Univerisity, Durham, NC, USA) [4,48], and UNC 2nd generation (The University of North Carolina at Chapel Hill, Chapel Hill, NC, USA) [32], as summarized in Table 1.

## 3. Experiment and Characterization

The multi-beam X-ray tube, integrating multiple focal spots within a single vacuum envelope, is the core component of the system. To meet sDCT requirements, the following design strategies are adopted: (1) enhancement of CNT field emission cathode performance to achieve higher current density and long-term stability at macroscopic emission areas, enabling high-current emission under longer pulse widths; (2) design of a multi-window X-ray extraction structure to achieve the required 36° angular coverage, 16.8° cone angle, and sufficient fan angle to fully cover the detector; (3) development of focusing structures to confine each electron beam and reduce focal spot size for high-resolution medical imaging.

### 3.1. CNT Field Emission Cathode

**Figure 4:**
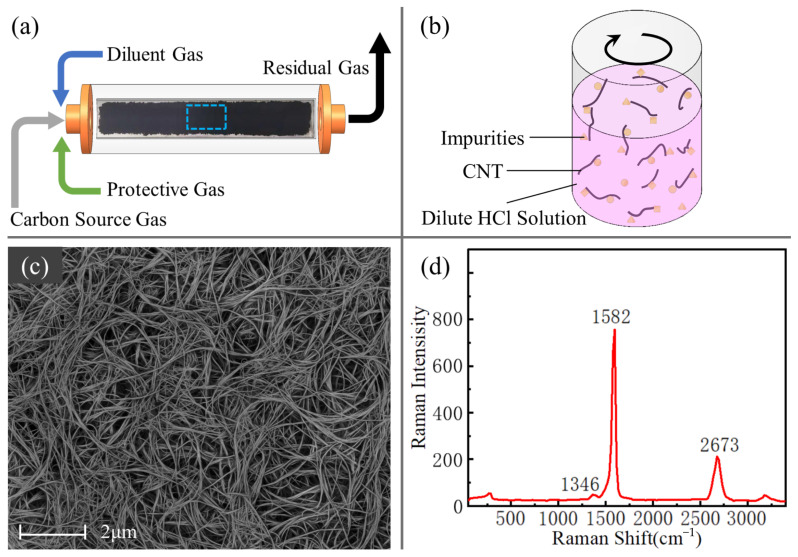
Preparation and characterization of few-walled CNTs: (**a**) schematic illustration of CNT growth; (**b**) schematic illustration of chemical purification; (**c**) SEM image of the CNTs; (**d**) Raman spectroscopy results.

To improve the cathode emission current density and long-term operational stability, few-walled CNTs were synthesized in this study by employing the chemical vapor deposition (CVD) method, as shown in Figure 4a. Regions with relatively superior growth quality were selected through physical area screening, as indicated by the blue boxed area in Figure 4a, and were followed by chemical purification (Figure 4b). As a result, high-purity (>99%, Figure 4c), low-defect (defect density < 0.07, Figure 4d), few-walled CNT materials were obtained.

**Figure 5:**
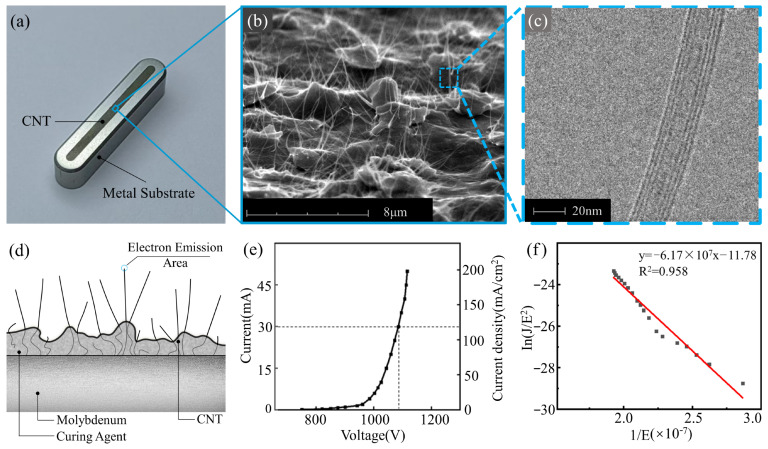
CNT cathode morphology and performance test: (**a**) physical image of the CNT cathode; (**b**) SEM image of the cathode surface; (**c**) TEM image of the CNTs; (**d**) schematic cross-sectional view of the cathode; (**e**) I–V curve of the cathode; (**f**) F–N curve of the cathode.

Few-walled CNTs were subsequently deposited onto molybdenum substrates using electrophoretic deposition combined with a surface solidification process to fabricate CNT cathodes. This process enabled the formation of a relatively uniform and well-dispersed CNT emission layer with locally aligned protruding CNT structures on the cathode surface, as shown in Figure 5.

Figure 5a shows a photograph of the fabricated CNT cathode, in which the CNT emission region exhibits a strip-shaped geometry. Figure 5b presents a scanning electron microscope (SEM) image of the cathode surface, revealing a moderately rough surface morphology with relatively uniform CNT distribution and locally protruding surface features. Figure 5c shows a transmission electron microscope (TEM) image of the few-walled CNTs, where a three-walled structure can be clearly observed. Figure 5d illustrates a schematic cross-sectional view of the cathode structure, in which the curing agent enhances the mechanical bonding between the CNTs and the molybdenum substrate. CNTs located at surface protrusions are more likely to experience local electric-field enhancement and therefore preferentially initiate electron emission under the applied electric field. Figure 5e shows the I–V characteristics of the CNT cathode. At a gate voltage of 1085 V, an emission current of 30 mA was achieved. Figure 5f shows the F–N curve derived from the I–V measurement results of the CNT cathode.

The operating principle of CNT cathodes has been extensively studied. Owing to the high aspect ratio of CNTs, strong field enhancement is achieved at the tips under moderate applied electric fields, enabling efficient electron emission without the need for high external fields. The emitted electrons from numerous tips collectively form the cathode electron beam.

According to the Fowler–Nordheim (F–N) field emission theory equation [49]:

(1) $$J=\frac{AE^{2}}{\emptyset }\exp\left(-\frac{B\emptyset^{\frac{3}{2}}}{E}\right)$$

After transformation, the equation can be written as

(2) $$\ln\left(\frac{J}{E^{2}}\right)=-B\emptyset^{\frac{3}{2}}\frac{1}{E}+\ln\left(\frac{A}{\emptyset }\right)$$

where *J* is the current density, *E* is the electric field strength, and ∅ is the work function of CNT; here, ∅ = 5 eV, *A* = 1.5414 [µA][eV][V]^−2^ and *B* = 6.83089 [eV]^−3/2^[V][nm]^−1^ are constants. Equation (2) indicates that ln(*J*/*E*^2^) has a linear relationship with 1/*E*, demonstrating that the cathode electron emission follows a field emission mechanism. The I–V curve shown in Figure 5e exhibits an exponential increasing trend, consistent with Equation (1). Furthermore, the F–N plot in Figure 5f shows good agreement with the theoretical prediction of Equation (2).

The fabricated CNT cathode was able to operate stably at an emission current of 30 mA for an extended period, corresponding to a current density of 120 mA/cm^2^ and a field enhancement factor of 1238. The vertically protruding CNTs observed in the microstructure are expected to experience enhanced local electric fields at their tips, which is beneficial for field electron emission. The CNTs are well-dispersed with sufficient spacing, reducing electrostatic shielding effects between neighboring emitters. Compared with previous designs, both the uniformity and density of CNT distribution are significantly improved, resulting in a high density of emission sites and consequently a large emission current.

### 3.2. Focusing Structure Design

Within the multi-beam X-ray source, each cathode–anode pair forms an array configuration. The electric field between them is approximately parallel, which can lead to beam divergence during electron transport and therefore requires a dedicated focusing design.

**Figure 6:**
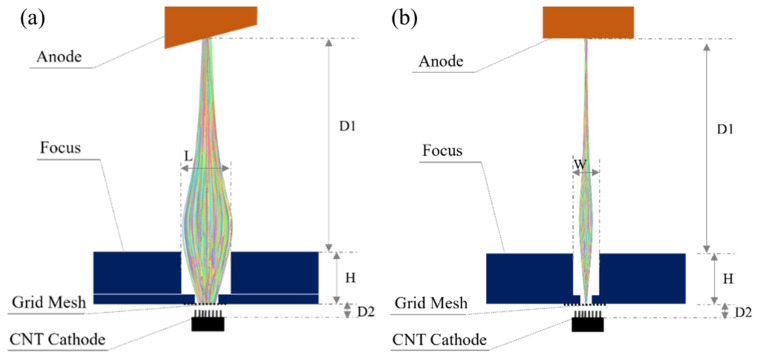
Schematic of the X-ray tube focusing structure. (**a**) Side of electron beam length; (**b**) side of electron beam width.

Key parameters for the focusing structure are determined based on the electron transport path from cathode to anode. The anode target angle is set to 10° to meet the required X-ray emission geometry. Under a gate voltage of 1500 V and an anode voltage of 120 kV, the electron beam transport is simulated using Opera 3D (version 18R1). Structural parameters, including focusing aperture length, aperture width, focusing electrode height, and cathode-to-anode distance (D1), are optimized to achieve a focal spot size smaller than 1.0 mm. When L = 21 mm, W = 10 mm, H = 10 mm, D1 = 18 mm, D2 = 0.215 mm, voltage between anode and ground Vag = 120 kV, and voltage between ground and cathode Vgc = 1.5 kV, the goal of focus spot size reaches less than 1 mm by Opera 3D simulation. The simulated focusing performance is shown in Figure 6, yielding a focal spot size of 0.856 mm × 0.959 mm.

**Figure 7:**
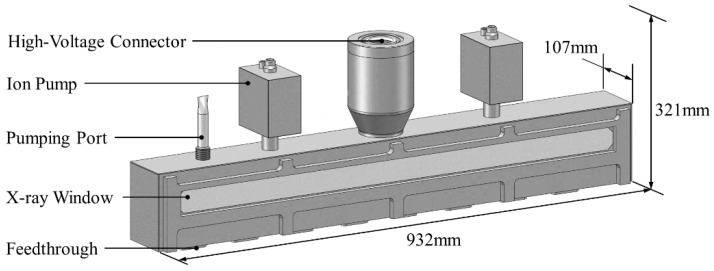
Overall design of the multi-window multi-beam X-ray tube.

Based on the above designs for the cathode, X-ray window, cathode–anode array, and focusing structure, the complete linear multi-beam X-ray tube assembly is realized. The tube envelope is fabricated from 3 mm thick stainless steel, while the X-ray windows use 0.2 mm stainless steel foil. The anode is supported within the vacuum chamber by ceramic insulation, maintaining sufficient electrical isolation while also serving as the high-voltage connection structure, compatible with standard R24 high-voltage cables. Independent control of 63 cathodes is achieved via 8 feedthroughs with 8 pins each. The tube also includes a vacuum port for leak testing and bake-out during sealing, as well as an ion pump to maintain high vacuum during operation. The overall structure and dimensions of the multi-beam X-ray tube are shown in Figure 7.

## 4. Results and Analysis

A linear multi-beam X-ray tube was fabricated and assembled according to the above design. After high-temperature bake-out and evacuation, the pumping port was pinch-sealed to maintain a high vacuum. A XRV160P4000 power supply (Spellman High Voltage Electronics Corporation, Hauppauge, NY, USA) was used to provide a positive anode voltage of 0–140 kV. A custom multi-channel electronic control system ECS-C096M (NuRay Technology Co., Ltd., Changzhou, China) was employed for constant-current control of CNT cathodes, enabling independent control of 63 cathodes with adjustable current from 0 to 30 mA and pulse width from 1 to 20 ms. After system integration, the tube was conditioned by gradually increasing the anode voltage and current, followed by performance characterization. Previous experimental verification demonstrated that when the X-ray tube operates under the conditions of 140 kV anode voltage, 30 mA cathode current, 20 ms pulse width, 20 ms pulse interval, and sequential operation of 63 focal spots, with a 5 min cooling interval after each scan, the X-ray source can operate normally without considering the influence of thermal load. Stable operation can also be maintained under conditions lower than these operating parameters.

### 4.1. Tube Voltage and Half-Value Layer

**Figure 8:**
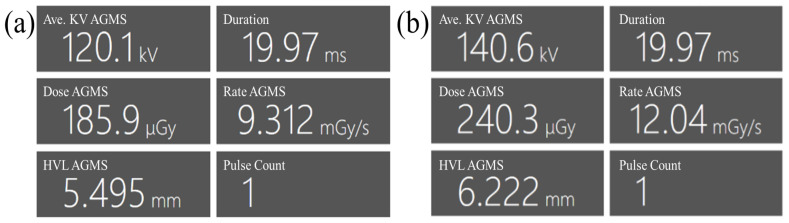
Tube voltage and half-value layer measurements. (**a**) Pulse parameters tested at 120 kV; (**b**) Pulse parameters tested at 140 kV.

The multi-beam X-ray source, consisting of a 63-focal-spot tube, high-voltage supply, and multi-channel control system, was evaluated using the central focal spot (#32). A Accu-Gold AGMS-D+ X-ray analyzer (Radcal Corporation, Monrovia, CA, USA) was positioned 50 cm in front of the focal spot. The source was operated at an anode current of 20 mA, pulse width 20 ms, and duty cycle 1%. Measurements were conducted at a nominal operating voltage of 120 kV and a maximum voltage of 140 kV. Results are shown in Figure 8.

As shown in Figure 8a, under nominal conditions, the measured anode voltage was 120.1 kV, consistent with the set value of 120 kV. The aluminum half-value layer (HVL) was 5.495 mm, corresponding to the higher end of the energy spectrum at 120 kV, indicating strong penetration capability. The measured pulse width was 19.97 ms, consistent with the set value of 20 ms, and the dose rate at a distance of 50 cm was 9.312 mGy/s. As shown in Figure 8b, when the voltage increased to 140 kV, the HVL increased to 6.222 mm, and the dose rate increased by 29.3%, indicating a significant enhancement in X-ray output, which is beneficial for improving the applicability and imaging performance of the sDCT system.

### 4.2. Anode Current Uniformity

**Figure 9:**
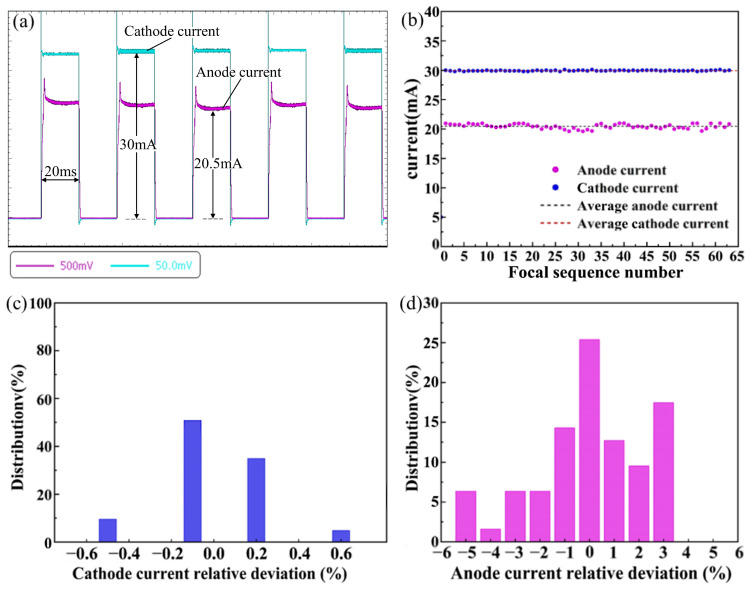
Consistency of cathode and anode currents for 63 focal spots. (**a**) Cathode–anode current waveforms; (**b**) corresponding cathode–anode current values; (**c**) analysis of cathode current variations; (**d**) analysis of anode current variations.

The multi-beam X-ray tube contains 63 focal spots, each with an anode current generated by its corresponding CNT cathode and controlled by the ECS-C096M (NuRay Technology Co., Ltd., Changzhou, China). The ECS regulates the cathode emission current I by adjusting the gate voltage V_gc_ of the CNT cathode. Meanwhile, the cathode emission current is continuously sampled and controlled through real-time feedback to maintain a constant emission current. As the operating time of the CNT cathode increases, the gate voltage V_gc_ gradually rises to compensate for the current degradation of the CNT cathode, thereby maintaining stable cathode current throughout operation. After the cathode current is generated, a portion is intercepted by the grid when passing through it, and the current that passes through the grid is further subject to a capture efficiency factor during focusing and anode bombardment. Therefore, the anode current depends on ECS control accuracy, grid transmission, and focusing/anode capture efficiency, leading to potential variation across focal spots. A Tektronix MDO3024 oscilloscope (Tektronix, Inc., Beaverton, OR, USA) was used to simultaneously monitor cathode and anode currents and evaluate consistency across 63 focal spots. Test conditions were: anode voltage 140 kV, cathode current 30 mA, pulse width 20 ms, pulse interval 20 ms, with 63 focal spots activated sequentially. Representative waveforms are shown in Figure 9.

As shown in Figure 9a, both cathode and anode currents exhibit slight overshoot at pulse onset, quickly stabilizing to a flat-top waveform, indicating constant-current operation. When all 63 cathodes were set to 30 mA, the average cathode current was 29.9 mA, and the average anode current was 20.5 mA, corresponding to an overall transmission and capture efficiency of 68.6% (Figure 9b). Under ECS control, cathode current variation ranged from −0.5% to 0.6%, with a standard deviation of 0.01 (Figure 9c), demonstrating high control precision. In contrast, anode current variation was larger (−5.5% to 3.5%), with a standard deviation of 0.39 (Figure 9d), due to the combined effects of grid transmission, capture efficiency, and secondary electron scattering.

In medical tomosynthesis imaging, the X-ray intensity produced by each focal spot, which corresponds to the anode current, needs to be highly consistent. This means that either the anode currents of all focal points should be uniform, or the X-ray intensity at the center of the imaging region for each focal point should be equivalent. The ECS developed in this study allows programmatic adjustment of the current for each cathode, enabling the X-ray intensity of each focal point to be set to a uniform target value. Spronk et al. [50] at UNC described a specific control method to achieve optimized imaging performance, which can similarly be applied to the 63-focal-spot multi-beam X-ray source developed here for sDCT applications.

### 4.3. Focal Spot Size Measurement

**Figure 10:**
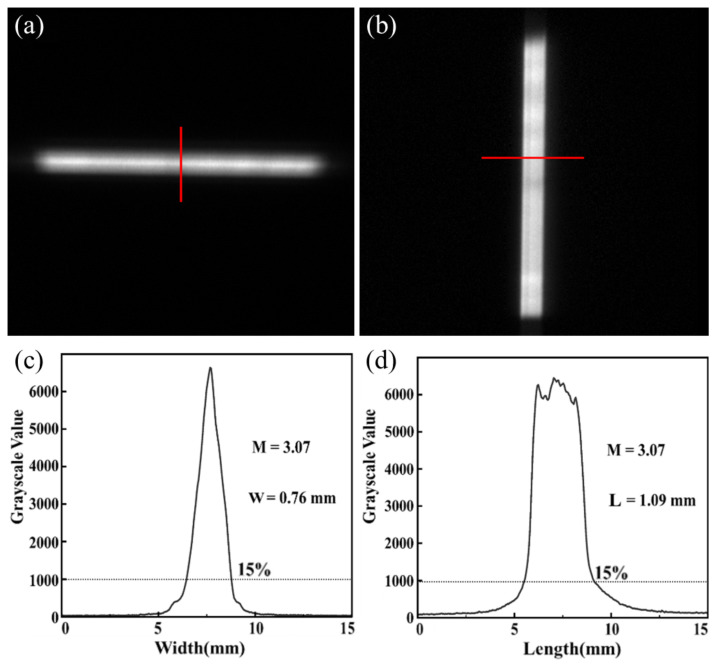
Focal spot size measurement for focal spot #23. (**a**) Slit image in the width direction; (**b**) slit image in the length direction; (**c**) gray value distribution along the width; (**d**) gray value distribution along the length.

Focal spot size is a critical parameter determining spatial resolution in medical imaging. Achieving both high emission current density and high cathode current enables reduction of the emission area, resulting in smaller focal spots. To measure the effective focal spot size after focusing and line-focus projection on a reflective anode, a slit camera using the Pro-Slit system (Pro-Project Group Sp. z o.o., Chelm, Poland) (discretization error: 1%) and a flat-panel detector, the Shad-o-Box 3K HS (Teledyne DALSA, Thousand Oaks, CA, USA) (CsI scintillator; pixel matrix: 2304 × 1300; pixel size: 49.5 μm), were employed in accordance with IEC 60336:2020 [51]. Twenty-one focal spots were measured; a representative result for focal spot #23 is shown in Figure 10.

Figure 10 shows the images of the focal spot projected through a slit onto the detector using the slit method, along with the corresponding gray-value distributions in the respective directions. The slit was placed between the X-ray window and the flat-panel detector, with a geometric magnification factor of M = 3.07. The focal spot size was determined using the 15% peak intensity criterion. The measured focal spot size is 0.76 mm (width) × 1.09 mm (length), corresponding to IEC 0.6. This relatively small focal spot provides a solid foundation for achieving high spatial resolution and superior image quality in X-ray imaging systems. Statistical results for 21 sampled focal spots are shown in Figure 10.

**Figure 11:**
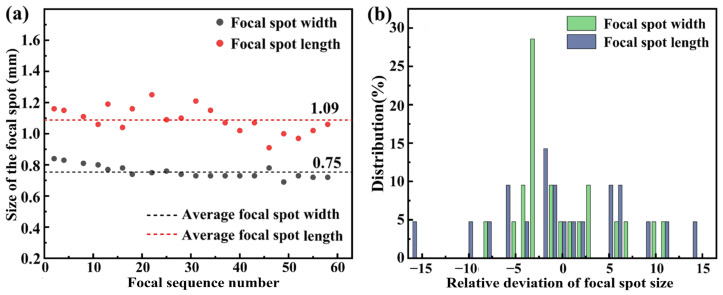
Test results for 21 evenly spaced focal spots. (**a**) Statistical results of focal spot sizes; (**b**) analysis of focal spot size variation.

As shown in Figure 11a, focal spot variations are attributed to differences in cathode emission characteristics and fabrication/assembly tolerances of focusing structures. The widths of 21 focal spots ranged from 0.69 to 0.84 mm (mean 0.75 mm), while the lengths of 21 focal spots ranged from 0.91 to 1.25 mm (mean 1.09 mm). As shown in Figure 11b, width variation is relatively small, whereas length variation is more dispersed, with a maximum deviation of 15%.

### 4.4. Cone Angle and Fan Angle Measurement

A key design objective is to ensure that X-rays from all focal spots, emitted through seven windows, fully cover the same detector and imaging region. This requires verification of both cone angle and fan angle for each focal spot.

The angular spread perpendicular to the focal spot array is defined as the cone angle, determined by the vertical aperture of the X-ray window. The angular spread parallel to the array is defined as the fan angle, determined by the horizontal aperture. In addition to geometric constraints, X-ray intensity distribution is affected by the heel effect in the cone direction [52] and by increased attenuation at large oblique angles in the fan direction due to longer path length through the window material [38]. To ensure acceptable uniformity for medical imaging, the usable field is defined on the detector plane as the region where X-ray intensity remains above 80% of the central beam intensity.

**Figure 12:**
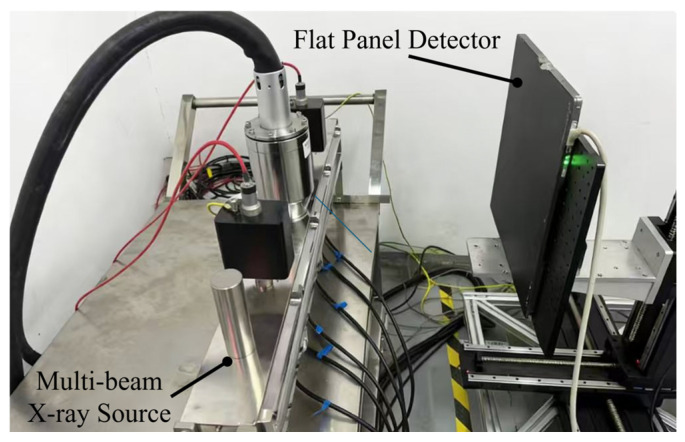
Cone angle and fan angle measurements.

A Mercu 1717V flat-panel detector (IRAY, Shanghai, China) (CsI scintillator; pixel matrix: 3072 × 3072; pixel size: 139 μm) was positioned along a line parallel to the focal spot array at 50 cm from the source, as shown in Figure 12. For each measurement, the detector was aligned directly in front of the focal spot under test to acquire X-ray intensity signals.

**Figure 13:**
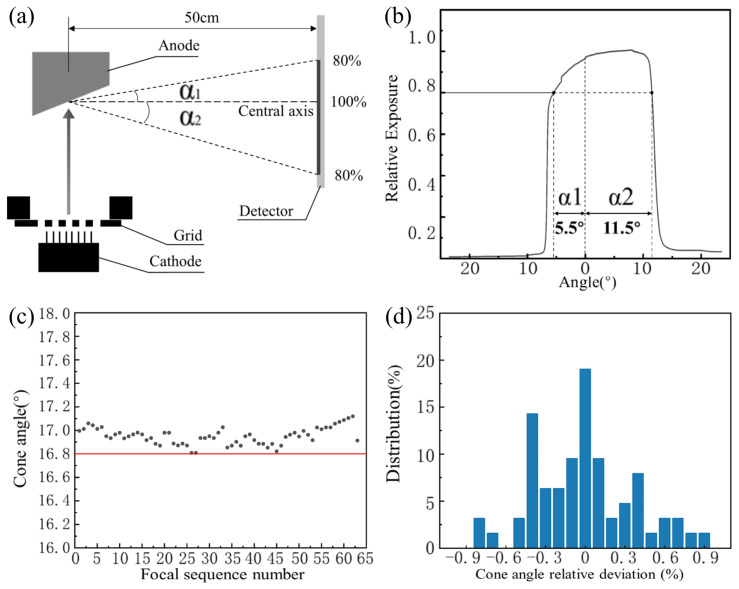
Cone angle measurement schematic and results. (**a**) Schematic of cone angle; (**b**) cone angle of focal spot #32; (**c**) cone angle measurement results; (**d**) distribution of cone angle variations.

During testing, the X-ray tube was operated at an anode voltage of 120 kV and an anode current of 20 mA. The detector plane was maintained perpendicular to the forward horizontal axis of each focal spot under test. For cone angle measurements, the center of the flat-panel detector was aligned with the focal spot position. The detailed measurement method and results are shown in Figure 13.

As illustrated in Figure 13a, when the detector center is aligned with the focal spot, the upper region corresponds to the upper cone angle α_1_ and the lower region to the lower cone angle α_2_. For the central focal spot (#32), the cone angle measurement is shown in Figure 13b. The pixel intensity profile along the vertical line through the detector center was plotted, and the angular coordinate was calculated by dividing the distance from the center by 50 cm (SDD) and applying tan^−1^ conversion. The measured upper cone angle was α_1_ = 5.5°, and the lower cone angle was α_2_ = 11.5°, yielding a total cone angle of 17.0°. This result is representative; measurements across all focal spots are summarized in Figure 13c, with an average value of 17.0°, all exceeding the design requirement of 16.8°. The variation among focal spots (Figure 13d) lies between −0.8% and 0.9%, with a standard deviation of 0.07, which is within measurement uncertainty.

**Figure 14:**
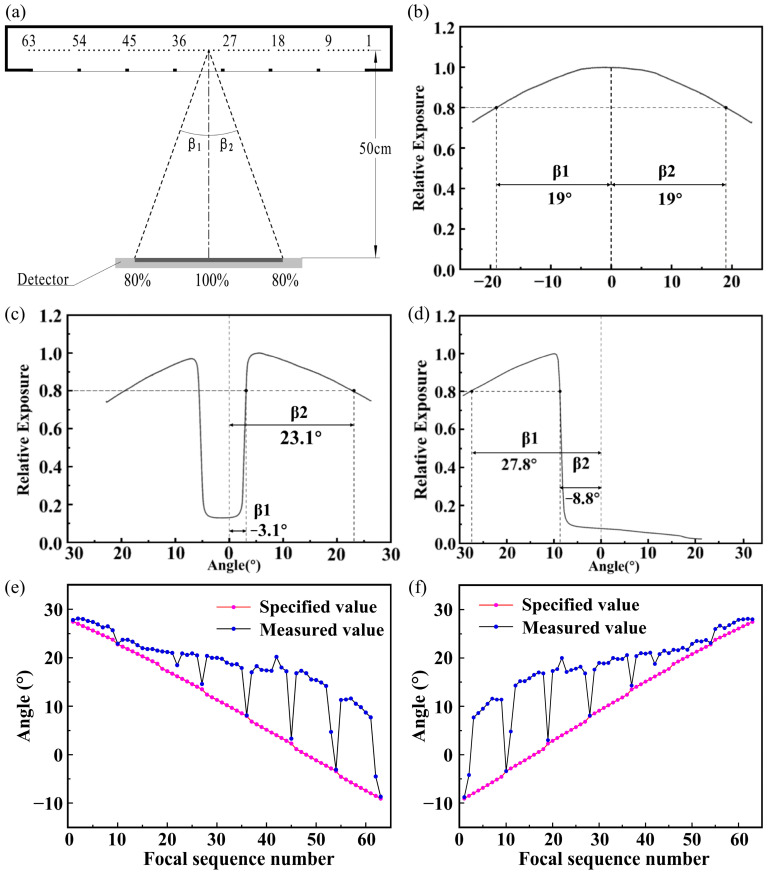
Fan angle measurement schematic and results. (**a**) Schematic of fan angle measurement; (**b**) fan angle results for #32; (**c**) fan angle results for #54; (**d**) fan angle results for #1; (**e**) results of left fan angle β_1_; (**f**) results of right fan angle β_2_.

During fan angle measurement, the detector center is aligned with the focal spot under test. The fan angle on the left side of the central axis is defined as β_1_, taken as positive when it lies on the left side, and negative if, due to obstruction from the supporting structures between X-ray windows, it extends to the right side of the central axis. Similarly, the fan angle on the right side is defined as β_2_, taken as positive when it lies on the right side, and negative if it extends to the left side due to such structural obstruction, as shown in Figure 14a.

Fan angle measurements were performed simultaneously with cone angle measurements. The pixel intensity profile along the horizontal line through the detector center was plotted, and angular coordinates were derived using the same tan^−1^ method with 50 cm (SDD). For focal spots without structural obstruction, the results are shown in Figure 14b, where for focal spot #32, β_1_ = 19° and β_2_ = 19°. For focal spots partially blocked by support structures, asymmetric fan angles were observed: for focal spot #54 (Figure 14c), β_1_ = −3.1° and β_2_ = 23.1°; for focal spot #1 (Figure 14d), β_1_ = 27.8° and β_2_ = −8.8°. Compliance is determined by whether both β_1_ and β_2_ exceed the design thresholds to ensure full detector coverage. Comparisons between measured and design values for β_1_ and β_2_ are shown in Figure 14e and Figure 14f, respectively. All focal spots meet or exceed the design requirements, ensuring full coverage of a detector area of 35.6 cm × 43.2 cm. It is also observed that focal spots near window edges have fan angles just meeting requirements on the constrained side, while other directions often exceed the requirements, resulting in some unnecessary X-ray output.

## 5. Discussion

This study addresses the limitations identified by UNC [32], where the dose and mAs of second-generation sDCT systems remain below those of current commercial systems by significantly improving the pulsed power performance of CNT-based multi-beam X-ray sources. In addition, key parameters such as maximum anode voltage (kVp) and focal spot size have been upgraded. Experimental results confirm that the design targets listed in Table 1 are achieved.

### 5.1. Field Emission Performance

A key improvement of the proposed X-ray source array is the enhancement of field emission performance, including emission current magnitude, sustainable pulse width for single operation, consistency among different cathodes, and long-term operational stability.

In this study, the purity of the few-walled CNTs was further improved through the physical region-selection and chemical purification processes shown in Figure 4. In addition, the electrophoretic deposition and surface-curing processes were optimized to improve the surface morphology of the CNT cathode, enabling the CNTs to exhibit better dispersion, uniformity, and vertical alignment. These improvements allowed the CNT tips located at protruding peak regions to obtain stronger local electric-field enhancement effects, thereby increasing the emission current.

Experimental results demonstrated that a cathode emission current of 30 mA could be achieved at a gate control voltage of 1085 V, corresponding to an anode current of 20.5 mA, which represents one of the highest reported levels for multi-beam CNT cold-cathode X-ray sources. Furthermore, based on these improvements, the pulse width of the emission current was increased from 2 ms to a maximum of 20 ms while maintaining stable constant-current operation, as shown in Figure 9a, demonstrating the excellent field emission performance of the few-walled CNT cathode.

Benefiting from the increased emission current and pulse width, a new sDCT system based on this X-ray source array can achieve operating parameters of up to 0.4 mAs per view and 24 mAs per scan, which are closer to those of commercial scanners. Since sDCT eliminates mechanical X-ray tube motion, motion artifacts can be avoided, and it is therefore expected that better image quality may be achieved at the same mAs level. However, the optimal mAs range for achieving the best sDCT image quality still requires further validation through phantom imaging experiments and image-quality evaluations. The capability of the proposed X-ray source array to provide up to 24 mAs offers a significantly broader operating range for future sDCT systems.

Field emission characteristics inevitably vary among different CNT cathodes. In this work, a specially developed electronic control system (ECS) was used to effectively equalize the emission currents of different cathodes to 30 mA, as shown in Figure 9b. Furthermore, the anode currents could be further equalized through differentiated cathode-current settings [50]. This technique effectively suppresses the influence of cathode-to-cathode variation. Meanwhile, the ECS continuously adjusts the gate control voltage through a real-time feedback mechanism, which can also compensate for emission degradation during long-term cathode operation.

The present study did not complete a systematic long-term stability evaluation of the CNT cathodes, although this characteristic is critically important for practical applications. While our recent study [49] demonstrated the long-term stable operation capability of CNT cathodes, the stability performance still needs to be experimentally verified for the complete X-ray source array presented in this work. This will be one of the major focuses of our future research.

### 5.2. Focal Spot Size

With improved focusing design (Section 3.2), the focal spot is reduced from IEC 0.8 to IEC 0.6, aligning with mainstream commercial systems such as Agfa DR-600 [42], Carestream DRX-Evolution Plus [45,46], Fujifilm FDR Visionary Suite [47], and GE Definium 8000 [41]. Some systems, such as Shimadzu SONIALVISION [43], further reduce focal spot size to IEC 0.4, improving spatial resolution but limiting tube power and mAs (e.g., 0.04 mAs per view). In the present study, the performance of the CNT cathodes has been significantly improved. While achieving an anode current of 20 mA, the cathode emission area still retains the potential for further reduction through optimization, thereby enabling additional reduction of the focal spot size. If future sDCT system development indicates that lower mAs is sufficient, the focal spot could be further optimized to IEC 0.4.

More importantly, sDCT systems based on the proposed X-ray source array eliminate motion artifacts caused by source movement and therefore have the potential to achieve higher spatial-resolution imaging. In conventional DCT systems employing mechanically moving X-ray sources, the focal spot continuously moves during the acquisition of each view, which is effectively equivalent to enlarging the focal spot size along the motion direction. Consequently, the IEC 0.6 focal spot provided by the present X-ray source array is expected to achieve better imaging performance than the IEC 0.6 focal spot in conventional moving-source DCT systems.

We plan to further validate this advantage through comparative imaging experiments in future work. Moreover, if the focal spot size can be further optimized to the IEC 0.4 level, the spatial-resolution advantage of sDCT imaging could be further enhanced.

### 5.3. X-Ray Irradiation Field

The X-ray irradiation field depends on the size of the diagnostic target object. In general, a detector slightly larger than the target object is selected, and the detector size subsequently determines the required X-ray irradiation field. Based on the spatial geometry between the detector and the X-ray source, the required cone angle and fan angle for each focal spot can then be calculated. When the focal spots of the X-ray source are arranged along the fan-angle direction, the cone angles of all focal spots remain identical, whereas the required fan angles vary sequentially among different focal spots, as illustrated by the line graph in Figure 14e,f of Section 4.4.

Due to the need to accommodate X-ray emission from a large number of focal spots, multi-beam X-ray sources impose significant structural constraints on the tube envelope. Early designs [37,38,39] predominantly adopted a long-slot window configuration, which provides a large fan angle but a limited cone angle, making it insufficient for medical imaging applications. As a result, the detector widths used in studies by J. Shan [36] and Billingsley [32] were limited to 30 cm. Experimental results in Section 4.4 demonstrate that this structure ensures high beam output accuracy and supports reliable X-ray emission over wider angular ranges. In future iterations, the cone angle is expected to be further increased beyond 20.4°, allowing the use of larger detectors (43.2 cm × 43.2 cm) at an SID of 120 cm, thereby better meeting the requirements of diverse medical imaging applications.

A multi-beam X-ray source with a multi-focal-spot array is essential for sDCT. However, in terms of X-ray emission through the window structure, both the earlier long-slot design and the multi-window approach proposed in this study share a common limitation: multiple focal spots emit through the same window. Although this enables dense focal spot distribution, it also results in fan angles that significantly exceed the detector coverage for many focal spots. Moreover, due to the close spacing between focal spots, it is difficult to implement individual collimation for each beam outside the X-ray window. As a result, a portion of the emitted X-rays irradiates regions outside the target anatomy, necessitating additional shielding to reduce unnecessary exposure. To further reduce patient dose, future designs may consider lowering focal spot density, increasing the spacing between adjacent focal spots, and reducing the total number of focal spots. While this may impact depth resolution in tomosynthesis imaging [28], depth resolution is primarily determined by the total angular coverage, where multi-beam X-ray sources have inherent advantages. Maintaining a large angular span while reducing the number of focal spots, combined with effective per-beam collimation, may provide a better balance between image quality and dose efficiency. This trade-off requires further investigation.

### 5.4. Imaging Testing and Phantom-Based Validation

Since the construction of the sDCT system is still ongoing, only the development and validation of a CNT cold-cathode multi-beam X-ray source for sDCT have been completed to date, achieving the target specifications for a mobile sDCT system. However, image quality assessment using real chest phantoms has not yet been performed. Therefore, it is not yet possible to verify whether the predefined parameters of the X-ray source array are fully optimal or appropriately configured, and further optimization remains necessary. Similarly, the clinical advantages of sDCT cannot be confirmed until a functional prototype is completed, approved for clinical testing, and compared against conventional motion-based DCT systems in terms of reconstructed image quality.

The next step is to integrate a high-frame-rate flat-panel detector (e.g., Shark4343FPI, frame rate 20 fps). With a pulse width of 20 ms per view and an interval of 30 ms for data transfer, a scan comprising 60 views can be completed within 3 s, significantly shorter than current commercial systems, thereby reducing breath-hold duration and motion artifacts. In practice, the total scan time of sDCT is largely limited by the detector frame rate. Further improvements in detector performance could enable even faster acquisition, potentially eliminating the need for breath-holding altogether.

After the prototype system is constructed, systematic evaluations will be conducted to assess workflow feasibility and operational stability. These evaluations will focus on the compatibility between the X-ray source array and the detector, as well as long-term operational stability, with particular emphasis on verifying the stable working duration of the X-ray source array within the sDCT system. In addition, chest phantoms will be used to perform imaging experiments at different focal spot positions and under varying mAs settings. The influence of mAs on image quality will be investigated to determine optimal exposure parameters. Furthermore, tomographic reconstruction algorithms will be developed and refined to generate sDCT-based tomographic images, enabling comprehensive evaluation of imaging performance and supporting the development of a next-generation sDCT prototype system.

## 6. Conclusions

This study addresses key limitations of multi-beam X-ray sources used in sDCT, particularly insufficient dose output and mAs. Starting from the system-level design of an sDCT configuration, the performance requirements of the multi-beam X-ray source were analyzed, and the single-pulse dose output was substantially improved. Key advancements include optimization of CNT field emission cathode technology to enhance stability under high-current, long-pulse operation; improved focusing design to reduce focal spot size; and an innovative multi-window structure that minimizes deformation of the X-ray window.

Experimental results demonstrate that the developed CNT-based multi-beam X-ray source integrates 63 focal spots along a linear span of 816 mm, with a maximum operating voltage of 140 kV, enabling thoracic imaging across a wide range of patient body types. The focal spot size is reduced from IEC 0.8 to IEC 0.6, and the anode current per focal spot reaches 20 mA, representing a leading performance level among similar technologies. With a maximum pulse width of 20 ms and up to 0.4 mAs per view, the system achieves performance comparable to high-end commercial DCT systems and contributes to improved image quality in sDCT systems.

When combined with a flat-panel detector of size 35.6 cm × 43.2 cm and frame rate 20 fps at an SID of 120 cm, the system can achieve an angular coverage of 36° and reduce total scan time to 3 s. This shortens patient breath-holding requirements, reduces motion artifacts, and further improves both in-plane and depth resolution.

Although the proposed multi-beam X-ray source had already demonstrated strong performance, further optimization remains possible. Continued improvements are expected to facilitate the transition of sDCT from a proof-of-principle system towards full device-level development. The capability of fast image acquisition has the potential to enable more reliable clinical diagnosis of thoracic diseases.

## Figures and Tables

**Table 1:** Target parameters for sDCT and comparison with similar devices.

	Agfa	Carestream	Fujifilm	GE	Shimadzu	Duke	UNC 2nd	This Project
X-ray energy (kV)	120 (Max 150)	Not specified (Max 150)	Not specified (Max 150)	120	120	120	120	120 (Max 140)
Tube current used (mA)	Not specified (Max 500)	Not specified (Max 1000)	Not specified (Max 1000)	125	25–200	Not specified	20.4	20
Pulse width (ms/view)	Not specified	Not specified	Not specified	2	1.6–2.5	Not specified	2	≤20
mAs per scan	≤16	Not specified	Not specified	15	2.96	5	2.34	≤24
mAs per view	≤0.53	Not specified	Not specified	0.25	0.04	0.07	0.039	≤0.4
Angular span (degrees)	15	30	27 (10–60)	30	40	20	35	36
views	30	61	≤60	60	74	71	60	60
FSS (mm)	0.6/1.2	0.6/1.2	0.6/1.2	0.6	0.4	0.6	0.8	0.6
Detector size (cm)	35.6 × 43.2	35 × 43/43 × 43	43 × 43	41 × 41	43.2 × 43.2	41 × 41	30 × 40	35.6 × 43.2
Detector framerate (fps)	5.2	4.5/9	Not specified	~5.3	15	6.4	10	20
SID (cm)	115	110/180	Not specified	180	110	183	130	120
Scan time (s)	5–10	7.6/15.2	4–12	11.3	5	11	6	3

## Data Availability

All data used in this study are available upon request from the corresponding author.
